# Drought Analysis in East Nusa Tenggara (Indonesia) Using Regional Frequency Analysis

**DOI:** 10.1155/2021/6626102

**Published:** 2021-04-10

**Authors:** Heri Kuswanto, Anggi Wahyu Puspa, Imam Safawi Ahmad, Fausania Hibatullah

**Affiliations:** ^1^Center for Disaster Mitigation and Climate Change, Institut Teknologi Sepuluh Nopember, Jalan Raya ITS, Surabaya 60111, Indonesia; ^2^Department of Statistics, Institut Teknologi Sepuluh Nopember, Jalan Raya ITS, Surabaya 60111, Indonesia; ^3^Departement of Actuarial Science, Institut Teknologi Sepuluh Nopember, Jalan Raya ITS, Surabaya 60111, Indonesia

## Abstract

Drought is a condition of a shortage of water that has an impact on economic activity. This research studies the severe drought area in Indonesia using Regional Frequency Analysis (RFA), based on daily precipitation data recorded at nine stations. The analysis reveals five homogeneous regions, based on discordancy and heterogeneity tests. Furthermore, the *L*-moment approach is applied to investigate the regional distribution and suggests that the Pearson type III distribution is the distribution that best fits the five regions. This distribution is also used to calculate the regional growth curve that is employed in the drought analysis. The drought return period analysis, for conditions of 40% of normal rainfall, concludes that the region containing the Fransiskus Xaverius, Gewayantana, and Mali stations has the highest drought risk, indicated by the fastest return period estimate of 2 years and 4 months. Moreover, the extreme drought analysis shows that two of the regions have the potential to experience the return of extreme drought, with less than 20% of normal rainfall, in less than four years.

## 1. Introduction

Drought can be defined in a general way as a reduction in water supply or moisture that is temporarily significantly below the normal or expected volume for a certain period. Hydrological drought is indicated by a lack of water in the hydrological system, which includes rivers, lakes, and reservoirs. In the economic field, a drought means a lack of water that has a detrimental effect on economic activity [1]. Drought is a natural disaster that is considered important, especially in the tropical and sub-tropical regions of the world [2]. A short but intensive drought can cause significant damage [3]. Based on data from the National Disaster Management Agency (BNPB), East Nusa Tenggara (Nusa Tenggara Timur, hereafter denoted as NTT) province is one of the provinces in Indonesia that experiences a severe drought. The province is listed as the top priority in the region that is most vulnerable to drought. Among the districts and cities in East Nusa Tenggara province that have experienced severe droughts are Lembaka Regency, East Sumba, Rote Ndao, and Ngekeo [4]. At the beginning of September 2018, several districts in East Nusa Tenggara experienced extreme drought, with up to 60 days without rain [5]. Considering this fact, it is important to study the future pattern of the drought in NTT which will be useful for formulating proper adaptation and mitigation strategies to minimize drought risks. Therefore, this paper investigates hydrological drought in NTT. Most of the previous researches in NTT have been focused on adaptation strategy to drought event (see [6, 7] among others), and very few studies have been focused on predicting the future drought condition. Kuswanto et al. [8] predicted the drought event in NTT through investigating the active zones of sea surface temperature, while Inas et al. [9] focused on the future climate projection in a more general sense.

In describing hydrological drought, a popular and important characteristic is its duration or length, which is vital for drought planning and mitigation. An estimate of the frequency (i.e., return period) with which this characteristic recurs forms the basis for assessing the severity of droughts and associated water shortages [10, 11]. Moreover, the spatiotemporal quantification of the return period can be used for establishing the proper water demand and supply system and helps to overcome the challenges faced in the hydrometeorological regulations of reservoirs in the southwest coast as pointed out by [12]. The estimated return period of drought can be an early warning, enabling the negative risks of droughts that might happen in the future to be minimized.

Drought can be characterized by its frequency, intensity, or duration, or by people's vulnerability to its effects [13]. Proper drought management requires a knowledge of the expected frequency of rainfall in low amounts in certain regions and in various conditions of return periods. A probabilistic approach is commonly used to estimate the average return period for drought (see [14–17];). In this study, drought analysis is carried out using Regional Frequency Analysis (RFA). One of the advantages of RFA is its ability to produce estimates of drought return periods that can then be used for mapping the drought risk. Furthermore, the concept of regionalization in RFA enables the use of data from other stations in a region to estimate the quantile events for each station in that homogeneous region. The present study estimates the return period of drought events in East Nusa Tenggara through RFA, using daily rainfall data from nine rainfall measurement stations in the province from the period 2015 to 2017.

RFA has been extensively applied to estimate the return periods of various weather-related variables, such as those in [18–23]. The identification of homogeneous regions is carried out using cluster analysis, based on the latitude, longitude, altitude, and daily average rainfall recorded at each station. This method was chosen as it can classify regions by giving a balanced number of stations within the group [24]. Five different distributions that are commonly used in RFA are generalized extreme value (GEV), generalized logistic (GLO), Pearson type III (PE3), generalized Pareto (GPA), and generalized normal (GNO). These distributions can estimate the lower and upper quantile of the rainfall. Using the homogeneity among the stations within the group, it can be assumed that one rainfall station represents the area in which that station is located. In summary, the objectives of this study can be listed as follows: (1) to cluster regions in NTT based on the drought characteristics; (2) to estimate drought return period in NTT based on daily rainfall using RFA.

This paper is organized as follows. The data examined, as well as the methods used in the analysis, are described in the Materials and Methods section, and the paper continues with the Results and Discussion sections. The last section concludes the analysis.

## 2. Materials and Methods

### 2.1. Data

**Figure 1:**
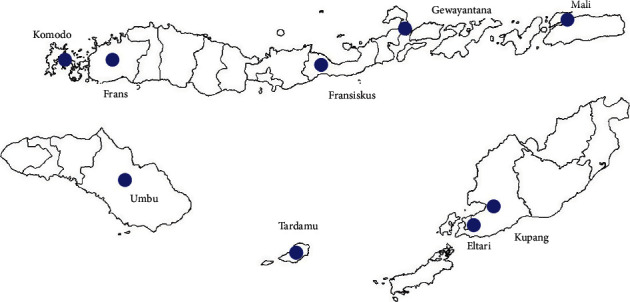
Geographical locations of stations in East Nusa Tenggara province.

This study uses daily rainfall data measured in millimetres per day for the period from 1 January 2005 to 31 December 2017 recorded at the following nine stations: Komodo, Frans Sales Lega, Fransiskus Xaverius, Gewayantana, Mali, Umbu Mehang Kunda, Kupang, Eltari, and Tardamu. The data were obtained from the official website of the Agency for Meteorology, Climatology and Geophysics (BMKG), which is available at http://dataonline.bmkg.go.id. Each station is assumed to represent the area of 250–1000 square kilometres around it. This refers to the standard rules for the density of rain stations in mountainous regions in tropical climate zones set by the WMO [25]. The spatial distribution of the stations can be seen in Figure 1.

As regards the geographical location of the island of East Nusa Tenggara (NTT), it is located between the continent of Asia and the continent of Australia, and between the Indonesian Ocean and the Flores Sea. NTT province consists of 21 districts and one city, located on seven major islands. The province has two seasons: a dry season and a rainy season. Because the province is close to Australia, wind containing a lot of moisture from Asia and the Pacific Ocean reaches the NTT region with reduced moisture, resulting in fewer rainy days in NTT than in other regions of Indonesia. For this reason, NTT is classified as a dry region, with only four wet months and eight dry months. The land use in East Nusa Tenggara province is dominated by agricultural activities, which cover an area of 214,714 hectares. The agricultural sector has become the highest contributor to Gross Regional Domestic Product (GRDP) in East Nusa Tenggara. This shows the significant role of the agricultural sector in NTT's economy [26].

The hydrological drought in the study is characterized by the drought events for between 10% and 50% of normal rainfall. Therefore, drought in this study is calculated from the percentage of rainfall from the MDP data which is below the normal condition.

### 2.2. Regional Frequency Analysis

Regional Frequency Analysis is an approach in which data from several sites are used to solve the problem of estimating the frequency of events at one site [24]. It involves the following main steps: (1) identification of homogeneous areas using cluster analysis; (2) data filtering using the discordance size *Di*; (3) homogeneity testing using the heterogeneity size *H*; (4) distribution selection using the *L*-moment ratio diagram and goodness-of-fit measure of goodness; and (5) estimation of quantile regional rainfall for a certain period of return [25].

### 2.3. Cluster Analysis

The configuration of homogeneous regions is based on cluster analysis, with site characteristic vectors being found for each site and standard multivariate statistical analysis being carried out on group sites according to vector similarity [24]. The variables used in the cluster analysis are latitude, longitude, altitude, and daily average rainfall. These variables are based on indicators of geographical location and rainfall indicators. Ward's minimum hierarchical variance classification framework is applied to the cluster analysis (Table 1). Ward [27] developed a hierarchical grouping procedure by minimizing the “loss of information” from a combination of two groups. This method is usually implemented with the “loss of information” taken as the increase in the number of squared errors, SSE. A combination of stations with the lowest SSE value forms one cluster/region. The SSE value can be calculated by the following formula:

(1) $$\begin{array}{c} \text{SSE}=\sum_{i=1}^{N}{\left(x_{i}-\bar{x}\right)}^{\prime }\left(x_{i}-\bar{x}\right), \end{array}$$

where **x**_*i*_ is multivariate size related to item *I* and $\bar{x}$ is the average of all items.

An alternative method used to determine the optimum number of clusters is the pseudo-*F* statistic formulated by [28]. The pseudo-*F* statistical formula is shown as follows:

(2) $$\begin{array}{c} \text{pseudo}-F=\frac{\left(R^{2}/\left(c-1\right)\right)}{\left(\left(1-R^{2}\right)/\left(n-c\right)\right)}, \end{array}$$

(3) $$\begin{array}{c} R^{2}=1-\frac{\text{SST}-\text{SSW}}{\text{SST}}, \end{array}$$

(4) $$\begin{array}{c} \text{SSW}=\sum_{i=1}^{n}\sum_{j=1}^{c}\sum_{k=1}^{p}{\left(X_{ijk}-\bar{X_{jk}}\right)}^{2}, \end{array}$$

where *X*_*ijk*_ is the characteristic of the *i*-th sample in the *j*^th^ and *k*^th^ groups, $\bar{X_{j}}$ is the sample average for the *j*^th^ variable, $\bar{X_{jk}}$ is the variable average for the *j*^th^ variable and *k*^th^ group, SST is the total sum of squares of the sample distance concerning the overall average, SSW is the total sum of squares of the square of the sample distance against the group average, *c* is the number of variables, *p* is the number of clusters, and *n* is the number of samples.

### 2.4. *L*-Moment

The *L*-moment is used to summarize the theoretical distribution of a sample observed from a random variable (*X*). Hosking and Wallis define the *L*-moment as a linear function of the Probability Weighted Moment (PWM), which is robust to outliers and not biased for small samples. The four *L*-moments associated with PWMs can be calculated as follows:

(5) $$\begin{array}{c} \lambda_{1}=\beta_{0},\\ \lambda_{2}=2\beta_{1}-\beta_{0},\\ \lambda_{3}=6\beta_{2}-6\beta_{1}+\beta_{0},\\ \lambda_{4}=20\beta_{3}-30\beta_{2}+12\beta_{1}-\beta_{0}. \end{array}$$

where (*λ*_1_) is a mean distribution (*L-*location) and (*λ*_2_) is a scale measure (*L-scale*) from the distribution. The *L*-moment ratio can be defined as follows:

(6) $$\begin{array}{c} \tau_{r}=\frac{\lambda_{r}}{\lambda_{2}},\;r=3,4,\ldots ,\\ L-C_{V}=t=\frac{\lambda_{2}}{\lambda_{1}},\\ L-C_{S}=t_{3}=\frac{\lambda_{3}}{\lambda_{2}},\\ L-C_{k}=t_{4}=\frac{\lambda_{4}}{\lambda_{2}}, \end{array}$$

where *L* − *C*_*v*_ · (*t*) = measure of variation, coefficient of *L*-variation, *L* − *C*_*v*_ · (*t*_3_) = measure of skewness, the coefficient of *L*-skewness, *L* − *C*_*k*_ · (*t*_4_) = measure of kurtosis, *L*-kurtosis coefficient.

### 2.5. Discordancy Measure

The discordancy measure (*D*_*i*_) aims to identify sites that are different from the whole group and is related to the L-moment ratio (*L* − *C*_*v*_, *L* − *C*_*s*_, *L* − *C*_*k*_) for a site in 3-dimensional space. The equations used in the calculation of discordancy size are as follows:

(7) $$\begin{array}{c} \bar{u}=N^{-1}\sum_{i=1}^{N}u_{i},\\ S=\sum_{i=1}^{N}\left(u_{i}-\bar{u}\right){\left(u_{i}-\bar{u}\right)}^{T},\\ D_{i}=\frac{1}{3}N{\left(u_{i}-\bar{u}\right)}^{T}S^{-1}\left(u_{i}-\bar{u}\right), \end{array}$$

where *N* is number of sites/rainfall measurement units in one group, **u**_**i**_ is a vector containing *t, t*_3_, and *t*_4,_**S** is covariance matrix sum of squares, *D*_*i*_ is discordancy.

Hosking and Wallis state that sites *i* with *Di* ≥ 3 can be considered unusual or different from the group as a whole (discordant) [21].

### 2.6. Heterogeneity Measure

This statistic is used to compare the homogeneity between sites in the region (measuring stations) to decide if they are homogeneous. Based on the simulation performed, the mean and standard deviation of the *V* values are determined as much as the number of simulation. The size of the heterogeneity can be calculated from the following formula:

(8) $$\begin{array}{c} H=\frac{\left(V-\mu_{V}\right)}{\sigma_{V}}, \end{array}$$

where *H* is the measure of heterogeneity, *V* is sample weighted standard deviation *L* − *C*_*V*_, *μ*_*V*_is the average of the *V* value of the simulation results, and *σ*_*V*_is the standard deviation of the *V* value of the simulation results. A region is said to be heterogeneous if the value of *H* obtained is large enough. A region is considered “acceptably homogeneous” if the value of *H* is less than 1, “possibly heterogeneous” if *H* is greater than or equal to 1 and less than 2, and “definitely heterogeneous” if *H* is greater than 2.

### 2.7. Regional Distribution Identification

The distributions examined are 3-parameter distributions, namely, generalized extreme value (GEV), generalized logistic (GLO), Pearson type III (PE3), generalized Pareto (GPA), and generalized normal (GNO). A test is carried out to select the appropriate distribution, that is, the distribution that matches the data, based on how the *L*-skewness and *L*-kurtosis of the fitted distribution correspond to the regional *L*-skewness and mean *L*-kurtosis of the recorded data.

### 2.8. Return Period

A return period is defined as the average time between events with at least a certain severity. In this study return, periods are calculated for drought events for between 10% and 80% of normal rainfall. The return period is calculated by determining the type of distribution that matches the data. The formula for a period return (*T*) is as follows:

(9) $$\begin{array}{c} E\left(\tau \right)=T=\frac{1}{p}, \end{array}$$

where *p* is the extreme probability of the distribution.

## 3. Results

### 3.1. Identification of Regions

The aim of the identification of regions is to form a group of stations that have homogeneous conditions, so that the frequency distribution of the stations is identical regardless of the factor scale of each station. This is usually achieved by dividing the existing stations into several groups through cluster analysis. In this research, the clustering process is carried out by grouping the nine existing stations into two, three, four, and five clusters. Furthermore, the optimum number of clusters is determined by the pseudo-*F* statistical values, as can be seen in Table 2.

**Figure 2:**
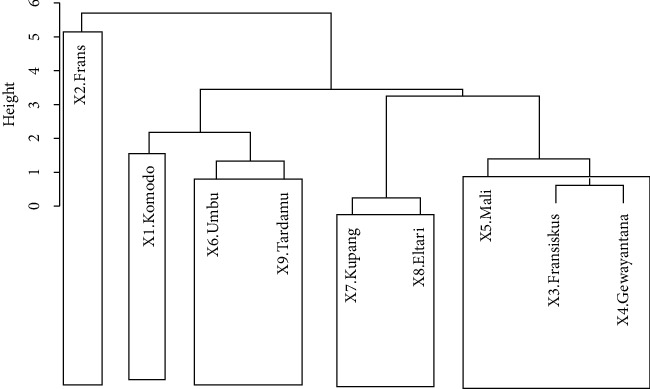
Dendrogram for grouping stations using Ward's method with five clusters. The clustering results follow the existing geographical conditions. This can be seen in the distribution of the clustering results in Figure 3.

Based on the results in Table 2, it can be seen that grouping the stations into five clusters produces the highest pseudo-*F* statistic, which suggests that the optimum number of clusters for the station grouping is five. The results for the initial clusters can be displayed in the form of a dendrogram, as shown in Figure 2.

Cluster 1 consists of Komodo station in the northwest, which has an average rainfall of 3.3 mm/day. Cluster 2 consists only of Frans Sales Lega station which has an average daily rainfall of 11.10 mm. Even though it is located near the Komodo station, Frans Sales Lega is part of a new cluster because it has different geographical characteristics and rainfall intensity. Cluster 3 consists of Fransiskus Xaverius, Mali, and Gewayantana stations, which are characterized by an average rainfall of between 3.16 mm/day and 3.78 mm/day. Cluster 4 consists of Umbu Mehang Kunda and Tardamu stations in the southwest, which have an average daily rainfall of between 2.51 mm and 3.4 mm. The last cluster consists of two stations in the southeast part of NTT, Kupang and Eltari. Each cluster is hereafter called a region.

### 3.2. Testing of Region Homogeneity and Selection of Regional Distribution

Before testing the homogeneity of the regions, the *L*-moment ratios (i.e., *L* − *C*_*v*_, *L* − *C*_*s*_, and *L* − *C*_*k*_), indicating the ratio of variance, skewness, and regional kurtosis, respectively, are calculated. The ratio becomes the basis for calculating the discordancy and heterogeneity statistics. The results of the cluster analysis need to be further investigated by looking at the discordancy and heterogeneity for the results of the initial clustering. More specifically, in a Regional Frequency Analysis, when a region has been identified as homogeneous, the discordancy size can be calculated for each station within the region. If a station is discordant or differs considerably from the other members of the region, then the possibility of moving the station to another region needs to be considered. The possibility of this subjective adjustment aims to improve the relationships and reduce the differences in a region, as measured by the heterogeneity measures. The purpose of the heterogeneity measurement is to estimate the degree of difference among a group of stations, and to assess whether the group of stations can be said to be homogeneous. The discordancy test results for each station in each region give a value for *D*_*i*_ less than the value of *D*_critical_ (3) as shown in Table 3. This means that no station deviates or conflicts with the other stations within its region. Furthermore, there are no results for the heterogeneity tests for regions 1 and 2 because both clusters only consist of one station each, so no comparison can be made and it cannot be said that the region is homogeneous/heterogeneous towards its members. For regions 3 and 4, the *H*1 values are 0.63 and 0.32, respectively, which means that regions 3 and 4 are “acceptably homogeneous,” because these values are less than 1. Region 5 has an *H*1 value of 1.83. This value is more than 1 but less than 2. Based on the decision-making category, it can be concluded that region 5 is “possibly heterogeneous.” In other words, the conclusion is that region 5 is homogeneous, but there is still a possibility that it is heterogeneous.

The next step is to determine the appropriate distribution for each region. The determination of the best-fit regional probability distribution can be done visually and by using statistical tests. Visual testing using the *L*-moment ratio diagram results in the conclusion that the regional *L*-moment point of each region fits the PE3 distribution curve. This is consistent with the results of the statistical analysis in Table 4. The results of the goodness-of-fit test suggest that PE3 is the best distribution to describe the daily rainfall data for all the regions.

### 3.3. Parameter Estimation and Regional Growth Curve

Table 5 displays the location (*μ*), scale (*σ*), and shape (*γ*) parameters of the PE3 distribution for each of the five homogeneous regions. These parameters are used to calculate the regional growth curve, based on the PE3 distribution function.

The estimation results for the regional growth curves for the five homogeneous regions are obtained from the PE3 distribution above. The regional growth curve value shows the magnitude of a very extreme event that has a 1/*T* chance of happening. Because drought is a condition in which the volume of the rainfall that occurs is less than the expected volume, this is indicated by a regional growth curve value that is less than 1. A regional growth curve value that approaches 0 indicates that a large number of extreme drought events may occur. For each regional growth curve, the probability of the exceedance value is used to calculate the return period.

### 3.4. Drought Return Period Analysis

Drought analysis in East Nusa Tenggara uses the length of the return period of drought. Drought in this case is defined as an event where the rainfall that occurs is 40% of the normal MDP. The drought limit of 40% of normal rainfall refers to the study conducted by [6]. The normal condition here refers to the average daily rainfall in each station within a region. Drought analysis is important for predicting the risk of extreme long-term drought in the agricultural sector and can be used as a mitigation tool. Table 3 shows that there is an increasing trend in *L* − *C*_*v*_ and *L* − *C*_*s*_ for the East Nusa Tenggara province to the east (from region 1 to region 3 and region 4 to region 5) except in region 2 (Frans Sales Lega) where *L* − *C*_*v*_ and *L* − *C*_*s*_ are lower than in the other regions. This is related to the same direction as the variability of the regional growth curves especially the right tail.

Concerning average rainfall, we see that the chances of very dry or wet events are greater in the eastern parts of NTT (region 5, and the surrounding areas) than in the western parts (region 1, region 2, and the surrounding areas). For instance, the 0.4 quantile from the regional growth curve (equivalent to 40% of the average normal rainfall event) in region 1 has a probability of exceedance of 0.3934. The calculation is carried out using quantile information and the probability of exceedance in Table 6 obtained from the interpolation.

This value is equivalent to a return period of around 2.5 years, calculated using equation (4). Furthermore, with the same calculation for region 3, the probability of exceedance is 0.4288, which is equivalent to a return period of around 2.33 years. This example proves that the eastern part of NTT (region 3) has a higher chance of becoming drier than the northwestern part (region 1). The drought return period of region 3 is shorter than that of region 1, which means that a drought in region 3 will recur faster than in region 1.

**Figure 4:**
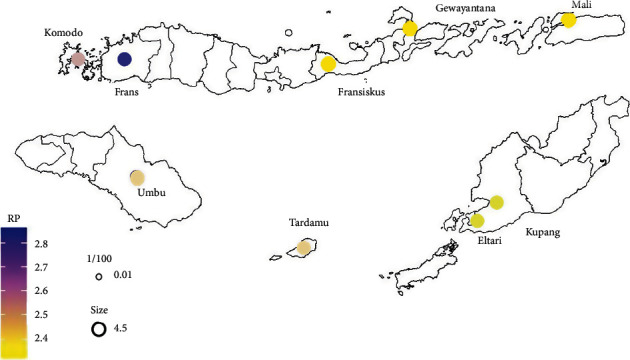
Mapping of drought return periods for 40% less rainfall than normal.

The return period value of other regions can be interpreted similarly. The results of the return period calculation are then mapped to define the pattern clearly. The mapping results can be seen in Figure 4, and it can be seen that the eastern parts of NTT (from Komodo to Mali) have lower return periods. The areas around region 3 (Fransiskus Xaverius, Gewayantana, and Mali) and region 5 (Kupang and Eltari) have return periods of about 2.3 and 2.4 years, respectively, indicating a higher probability of a faster recurrence of drought. Region 2 (Frans Sales Lega) is predicted to experience drought for several months longer than the other regions. In general, we observe that, in the eastern part of East Nusa Tenggara, the magnitude of droughts tends to increase and the drought return period is shorter.

The estimated return periods can be seen in Table 7, where, for a 50% drought, the estimated return period in each region tends to be the same (i.e., around 2 years 1 month). When drought is 40% to 20%, the estimated return period for each region tends to be longer, but with a length of less than five years. This means that within five years extreme drought can recur with less than 20% of the normal rainfall. In regions 3 and 5 in particular, extreme drought of less than 20% of normal rainfall recurs in less than four years, whereas for extreme drought conditions of 10% of normal rainfall, the estimated return period increases, and in region 1 it reaches 47 years and 2 months.

## 4. Discussion

The analysis above revealed that basically the regions in NTT can be grouped into five clusters with respect to the rainfall distribution as well as the location topology. If we look into more detail, the formed clusters are consistent with the spatial location of the region. Nevertheless, the distributions are similar but with different parameters, leading to different probability of exceedance, as can be seen in Table 6.

Table 7 reveals important information about the return periods on the corresponding region. The lower percentage means the drier the condition or more severe drought will happen. We see that the least severe drought (with 50% below normal) will recur about every two years. This finding is consistent with the study conducted by [29] who examined the climate scenario and found that the drought projection for Nusa Tenggara island shows that the severity of the drought that is less severe with a return period often occurs. Nevertheless, the level of severity is less than Bali and Java island. The results obtained in this study are also consistent with the finding of [30]. They used Standardized Precipitation Index (SPI) to characterize drought and found that Gewayantana is a district with the longest drought duration and strongest drought magnitude. Meanwhile, Komodo and Frans Sales Lega districts are two regions with the lowest risk indicated by shortest drought duration and lowest magnitude compared to the others.

In relation to the agricultural sector in East Nusa Tenggara, the estimated drought return period is useful as an early warning system in areas that have the potential for extreme drought. Although the results for the return period estimations for the five regions do not differ significantly, the two regions that have the greatest areas of agricultural land will experience high losses during drought events. The return period prediction allows farmers to formulate adaptation strategies for drought events to minimize losses due to crop failure. Mau et al. [31] studied ground nut productivity in NTT and found that unpredictable environ-mental conditions, especially the uncertain rainfalls, and water shortage have been the major limiting factors for groundnut production in this region. Unavailability of high yielding and well adapted varieties are additional limiting factors that cause yield of this crop in this region very low. Five-year records showed that groundnut productivity in NTT Province was only about 1.01–1.06 ton/ha of pod yield. In this research, the return period is estimated for drought conditions when the rainfall is 10%, 20%, 30%, and 50% of the normal amount, where a low percentage is associated with extreme drought conditions. Because extreme drought tends to result in greater losses for farmers who do not follow proper mitigation strategies, the results of this calculation will provide information to farmers about drought conditions in the corresponding region for better adaptation strategies. The estimated return period for extreme drought conditions can help farmers with their agricultural cultivation, so that the right action can be taken and the risk of loss can be minimized properly.

As part of the adaptation strategy, Climate Compatible Development (CDD) as discussed by [7] could be a solution on minimizing the drought risk. Priority strategies varied between the sub-districts but all reflected standard development interventions: water management, intensification or diversification of agriculture and aquaculture, education, health, food security, and skills-building for communities. The strategies have been well implemented in West Nusa Tenggara, which has similar climate characteristics to East Nusa Tenggara. This might be a big challenge due to the fact that the farmers' knowledge on projected weather and climate information is very poor [32]. Climate knowledge culture [6] which includes improving the stakeholder perspective would be an important factor as part of proper designing mitigation and adaptation strategies.

## 5. Conclusions

This paper discussed the future projection of drought events in East Nusa Tenggara by means of return periods. The nine stations in East Nusa Tenggara (Indonesia) can be grouped into five regions that are homogeneous and spatially close to each other. The fitting distribution indicates that the PE3 regional distribution is most suitable for the five regions. Furthermore, the fastest return period estimation for drought conditions is for 40% of normal rainfall. While the return period comparison for some extreme drought conditions shows a return period of fewer than five years, extreme drought can occur when rainfall is less than 20% of normal conditions. Region 3 and region 5 have the potential to experience extreme drought of 20% of normal rainfall less than four years after the previous event. This paper concludes that NTT will experience severe drought condition in the future; therefore, proper mitigation and adaptation strategies need to be well formulated. The results of this research are expected to provide useful information for policy-makers and farmers so that they can formulate proper adaptation strategies to deal with extreme drought.

## Figures and Tables

**Figure 3:**
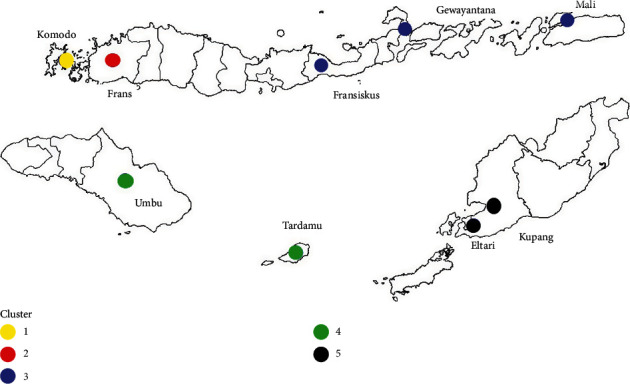
Location of each station based on cluster analysis.

**Table 1:** Variables used in the clustering analysis to find the homogeneous regions.

Variable	Information
Latitude	The latitude and longitude of the station
Longitude	

Elevation	The height of the station (metres above sea level)
MDP	Mean annual daily precipitation (mm)

**Table 2:** Pseudo-*F* statistics for two to five clusters.

Number of clusters	Pseudo-*F* statistic
2	7.28
3	6.86
4	10.35
5	14.21

**Table 3:** Estimated *L*-moment ratio, discordancy, and heterogeneity statistic for the daily rainfall average in the five homogeneous regions.

Region	Station (*D*_*i*_)	*L* − *C*_*v*_ · *R*	*L* − *C*_*s*_*R*	*L* − *C*_*k*_*R*	*D*_critical_	*H*_1_
1	Komodo	0.539	0.424	0.214	—	—
2	Frans Sales Lega	0.519	0.350	0.155	—	—
3	Fransiskus Xaverius (1), Gewayantana (1), Mali (1)	0.570	0.455	0.233	3	0.63
4	Umbu Mehang Kunda (1), Tardamu (1)	0.557	0.432	0.209	3	0.32
5	Kupang (1), Eltari (1)	0.568	0.442	0.225	3	1.83

**Table 4:** Goodness-of-fit test for rainfall data in the five homogeneous regions.

Region	Station (*Di*)	Best distribution (min |*Z*_dist_| 1.96)	|*Z*| (best fit)
1	Komodo	PE3	0.65
2	Frans Sales Lega	PE3	1.92
3	Fransiskus Xaverius, Gewayantana, Mali	PE3	1.02
4	Umbu Mehang Kunda, Tardamu	PE3	0.14
5	Kupang, Eltari	PE3	0.86

**Table 5:** Estimated regional distribution parameters of PE3 for the five homogeneous regions.

Parameter	Region				
	1	2	3	4	5
Location (*μ*)	1	1	1	1	1
Scale (*σ*)	1.156	1.052	1.255	1.204	1.234
Shape (*γ*)	2.563	2.103	2.768	2.616	2.674

**Table 6:** Probability of exceedance and return period for the five homogeneous regions.

Results	Region				
	1	2	3	4	5
Probability exceedance	0.3935	0.3507	0.4288	0.4102	0.4207
Return period (yrs)	2.5415	2.8518	2.3320	2.4379	2.3772

**Table 7:** Estimated rainfall return period for drought conditions of 10%, 20%, 30%, 40%, and 50% of normal rainfall.

% drought	Measure	Region				
		1	2	3	4	5
10% normal	Average rainfall	0.33	1.11	0.34	0.55	0.3
	*p*	0.0211	0.1131	0.06	0.0856	0.1026
	Return period	47 y 4 m	8 y 10 m	16 y 8 m	11 y 8 m	9 y 9 m

20% normal	Average rainfall	0.66	2.22	0.68	1.1	0.59
	*p*	0.213	0.2033	0.2569	0.2419	0.2559
	Return period	4 y 8 m	4 y 11 m	3 y 11 m	4 y 2 m	3 y 11 m

30% normal	Average rainfall	0.99	3.33	1.03	1.65	0.88
	*p*	0.3153	0.2811	0.3561	0.3369	0.35
	Return period	3 y 2 m	3 y 7 m	2 y 10 m	3 y	2 y 10 m

40% normal	Average rainfall	1.32	4.44	1.37	2.2	1.18
	*p*	0.3935	0.3507	0.4288	0.4102	0.4207
	Return period	2 y 6 m	2 y 10 m	2 y 4 m	2 y 5 m	2 y 5 m

50% normal	Average rainfall	1.65	5.55	1.72	2.75	1.47
	*p*	0.4571	0.4618	0.488	0.4701	0.4793
	Return period	2 y 2 m	2 y 2 m	2 y 1 m	2 y 2 m	2 y 1 m

Note: *p* shows the chance of an event returning; % drought shows the percentage of rainfall below normal conditions.

## Data Availability

The data were obtained from the official website of the Agency for Meteorology, Climatology and Geophysics (BMKG) which is available at http://dataonline.bmkg.go.id.
